# Cell therapy must be regulated as medicine

**DOI:** 10.1186/s40164-016-0055-0

**Published:** 2016-08-19

**Authors:** Zihai Li, Delong Liu

**Affiliations:** 1 Hollings Cancer Center, Medical University of South Carolina, Charleston, SC USA; 2 New York Medical College, Valhalla, NY USA

Cell therapy is as old as modern medicine. From blood transfusion to allogeneic hematopoietic stem cell transplant, cell therapy in the right settings has been proven to save lives. According to the American Red Cross, a total of 21 million blood components are transfused each year in the United States. The Center for International Blood and Marrow Transplant Research reports more than 50,000 patients worldwide were treated with hematopoietic stem cell transplantation annually.

Unfortunately, cell therapy is also a field that has a checkered history, which was plagued at one time or another by superstition, blatant medical malpractice, and lack of efficacies [1]. At one time blood transfusion from animals to humans was used as a punishment for human beings who were deemed evil and cadaver blood was once thought to be a lucrative commodity. Sadly, the discovery of blood types and the coagulation system was also associated with heavy tolls as countless lives were lost because patients received the “wrong” type or preparation of blood products. In the midst of these horrors and the devastating impact on human health, blood medicine pioneers learned the hard lesson that blood transfusion was vital but was not meant to be done by amateurs. As we improved our techniques in dealing with blood products, we were humbly reminded of how little we actually did understand about the natural law of life as transfusions of blood products could occasionally serve as a vehicle to transmit once considered fatal diseases such as HIV-1 infection and viral hepatitis.

Transfusion medicine has come a long way. It is now a vital medical specialty with its own well-established practices, principles and guidelines [2, 3]. It has cemented its place in the foundation of the modern medicine and blood therapy is now an undeniably effective and often the only treatment modality for a variety of conditions.

There are two fundamental roots to cancer: the cancer-intrinsic genetic or epigenetic dysregulation of the basic biology of the cell, and the failure of the cancer-extrinsic host immune defense mechanism known as immune surveillance to eliminate transformed cells [4]. Immunotherapy is particularly attractive because of its high specificity and the ability of the immune system to remember cancer cells and mounts a far more efficient immunological attack later when cancers recur. Not surprisingly, rejuvenating the host immune system for the treatment of cancer has been a dream pursued by generations of scientists. Presently, with technological advancements and a better understanding of the immune system, we are finally able to generate billions of T lymphocytes with defined antigen specificity, natural killer cells, as well as professional antigen-presenting cells such as dendritic cells [5]. Synthetic immunologists are especially proud of re-directing T cell antigen-specificity via enforcing the expression of chimeric antigen receptors and deleting the endogenous T cell receptor or other regulatory molecules such as program death 1 molecule (PD-1) via gene editing [6]. This has sparked a tremendous level of interests from the public for the miracle cures that immunotherapy might be able to deliver. Such a high level of enthusiasm was often further fueled by press releases and anecdotal reports of the wonder of killer cells in one form or another in animal studies or in early-phase clinical trials. Unfortunately, cell therapy for cancer is still at its experimental stage. The only US Food and Drug Administration (FDA)-approved cell therapy for cancer (excluding hematopoietic stem cell transplant), is sipuleucel-T which is indicated for the treatment of asymptomatic or minimally symptomatic metastatic castration-resistant (hormone refractory) prostate cancer [7]. Alarmingly, despite warnings from scientists, there is a widespread commercial use of cell therapy of unclear and unproven efficacy in cancer patients. This is particularly worrisome in countries where there is no clear-cut regulatory mechanism governing the practice of cell-based therapy for desperate cancer patients.

The exact number of patients who were given unproven cell therapy was unclear and might never be known, as the experience of these patients was rarely published in peer-reviewed international journals. It is less clear whether or not any of these patients had any meaningful clinical benefits and worse yet if more harms were done, medically or economically, to them. In examining some of the published work, it was often unclear if clinical studies were conducted under proper supervision by regulatory agencies and local institutional review boards. It was uncertain if these cellular products such as dendritic cells, or “cytokine-activated killer cells” that were administered to patients were manufactured under the principles of good manufacturing practice (GMP) with proper quality controls.

We believe that the application of cell therapy in human subjects under no clear scientific, ethical and regulatory monitoring by the pertinent agencies and policy makers is no better than an act of witchcraft. This practice is tainting the name of science, scientists or physicians alike. Worse yet, it puts the vulnerable public (i.e., the consumers or the patients) in a harm’s way. Lack of safeguard measures and quality controls to prevent adverse effects is unfortunately not uncommon. Since the outcome of the treatment is often not documented well and communicated to the scientific and medical community, the public is left without any analyzable scientific data to gain important knowledge to advance the medicine.

Encouragingly, the medical and research community is acutely aware of the lack of global standards in the practice of cell therapy and has been calling for actions. The US FDA continues to release guidance documents, describing the FDA’s current thinking on this topic (http://www.fda.gov/BiologicsBloodVaccines/GuidanceComplianceRegulatoryInformation/Guidances/CellularandGeneTherapy/). There is an ongoing debate elsewhere on whether cell-based therapy for cancer should be regulated as a medicine or can be merely regarded as a technology, which of course is under different jurisdictions. However, there is a high hope that the sweeping scientifically sound and responsible regulatory policy will be put in place in guiding cell therapy for cancer in both developed and developing countries. At the same time, unfortunately, many more patients continue to be treated by the experimental cell therapy without approval by the proper regulatory authorities; this include wide spread use of stem cells in non-cancer conditions [8–10]. For the sake of protecting patients, it is time now to halt such a practice until the appropriate regulatory policy is put in place. We believe in the power of science and by no means are calling for stopping proper clinical trials that unleash the wonder of immunology or stem cells. We are firmly convinced that meaningful conclusion cannot be drawn if clinical studies are not conducted correctly and ethically. In the case of unproven and unregulated commercial practice of cell therapy for cancer, it runs the risk of causing more harm than good to our patients, which we collectively have a moral and professional obligation to oppose.

## Abbreviations

CAHON: Chinese American Hematologist and Oncologist Network
FDA: The United States Food and Drug Administration
GMP: good manufacturing practices
HIV-1: human immunodeficiency virus-1
PD-1: program death 1 molecule (CD279)
